# Previously reported placebo-response-associated variants do not predict patient outcomes in inflammatory disease Phase III trial placebo arms

**DOI:** 10.1038/s41435-018-0018-z

**Published:** 2018-03-18

**Authors:** Asher Haug-Baltzell, Tushar R. Bhangale, Diana Chang, Amy Dressen, Brian L. Yaspan, Ward Ortmann, Matthew J. Brauer, Julie Hunkapiller, Jens Reeder, Kiran Mukhyala, Karen T. Cuenco, Jennifer A. Tom, Amy Cowgill, Jan Vogel, William F. Forrest, Timothy W. Behrens, Robert R. Graham, Arthur Wuster

**Affiliations:** 1 Genetics Interdisciplinary Group, University of Arizona, Tucson, AZ 85719 USA; 2 Department of Human Genetics, Genentech Inc, South San Francisco, 94080 CA USA; 3 Department of Bioinformatics and Computational Biology, Genentech Inc, South San Francisco, 94080 CA USA; 4 Biosample and Depository Management, Genentech Inc, South San Francisco, CA 94080 USA

**Keywords:** Genome-wide association studies, Autoimmunity

## Abstract

In clinical trials, a placebo response refers to improvement in disease symptoms arising from the psychological effect of receiving a treatment rather than the actual treatment under investigation. Previous research has reported genomic variation associated with the likelihood of observing a placebo response, but these studies have been limited in scope and have not been validated. Here, we analyzed whole-genome sequencing data from 784 patients undergoing placebo treatment in Phase III Asthma or Rheumatoid Arthritis trials to assess the impact of previously reported variation on patient outcomes in the placebo arms and to identify novel variants associated with the placebo response. Contrary to expectations based on previous reports, we did not observe any statistically significant associations between genomic variants and placebo treatment outcome. Our findings suggest that the biological origin of the placebo response is complex and likely to be variable between disease areas.

## Introduction

In clinical trials, the placebo response refers to a patient’s improvement in disease symptoms arising from the psychological effect of receiving a treatment rather than the actual treatment under investigation [1]. This improvement can be actual or perceived, and can encompass diverse causes such as the natural waxing–waning of disease symptoms, regression to the mean, response to concurrent medications, spontaneous remission, or a biologically mediated true placebo response [2, 3]. Considering that successful outcomes of placebo-controlled randomized trials are achieved by demonstrating a greater improvement in treatment arms than the placebo arms, and the primary cause of Phase III trial failure is a demonstrated lack of efficacy, it is clear that a solid understanding of the placebo response is essential for maximizing the chances of success of such trials [4, 5]. This is particularly important considering the current downward trend in clinical trial success rate, and a corresponding increase in cost per new drug approved that has, by some estimates, reached $2 billion [6].

One way to improve the chances of clinical trial success is by improving the trial design. A fundamental assumption underlying the placebo-controlled trial design is that placebo treatment serves as an effective and unbiased control, and thus failure to outperform a placebo arm is indicative of a lack of drug efficacy. However, extensive research highlighting the powerful and biological nature of the placebo effect calls this assumption into question [2, 7–9]. Neuroimaging studies have demonstrated that the placebo response can, at least in certain cases, be biologically mediated through the dopaminergic, opioid, serotoninergic, and endocannabinoid signaling pathways [3, 7]. These findings highlighted a possibility for uncovering the genetic basis of the placebo effect, the “placebome”, and identifying genomic variation which might be predictive of an individual’s propensity to experience the placebo effect [3, 7]. In addition, these findings suggest a possibility for an interaction between placebo pathways and a drug’s molecular pathway, which, if known, would have to be considered in the design of the study. A number of studies have been conducted to investigate the genetics of the placebo response, and have reported multiple variants that appear to be associated with the likelihood of an individual responding on placebo treatment under analgesic or psychiatric treatments [7]. Perhaps the best understood of these proposed variants is rs4680, a variant associated with an increased placebo response rate. The amino acid change (Val158Met) resulting from this variant causes a 3–4-fold reduction in the activity of Catechol-*O*-methyl transferase (COMT), thus increasing the brains natural analgesic ability, a logical explanation for the increased placebo response experience [10]. The reports that specific genetic variants may impart a stronger placebo-response phenotype suggest a potential for trial failure due to inadequately matching likely placebo responders between trial arms, and highlights a possible area for future trial improvement [11].

Although these studies have applied a variety of methodologies toward gaining a better understanding of the placebome, the genetic basis of the placebo response is not yet well characterized. For example, previous work has focused on a limited number of pain-related or psychiatric conditions, and thus may not be applicable more broadly to other diseases [3, 7]. Additionally, many of these studies have failed to replicate previous findings, further raising questions about their general applicability [7]. Despite the fact that common immune-mediated diseases make excellent models for inquiry into the nature of the placebo response, since they have well-characterized physiological features, established measurements of clinical status that are objective and easily obtained, and have been shown to respond to placebo treatment in a robust and objective manner, little to none of our current understanding of the genetic basis of the placebo effect has been derived from studies using these diseases [12–14]. To address this knowledge gap, we leveraged the genome-sequencing data from nine placebo-controlled randomized trials for two immune-mediated diseases, Asthma and Rheumatoid Arthritis (RA) [15–21]. Our goal was not to simply replicate previous work, but to instead assess the impact of the previously reported placebo response Single Nucleotide Polymorphisms (SNPs) on patient outcomes in the placebo arms of immune-disease trials, and to search for novel variants associated with the placebo response.

Measuring the placebo response can be challenging, especially when attempting to compare multiple different diseases. In this study, we chose to primarily focus on patient-reported wellness outcomes as reported in quality-of-life (QOL) questionnaires collected at the preliminary screening and regularly throughout the trial progression. Using these measurements, we classified patients dichotomously as either placebo “responders” (those who showed an improvement in QOL) and placebo “non-responders” (those who showed no improvement or a worsening of QOL). The choice of using change in QOL as the primary outcome measure was deemed necessary and appropriate for two major reasons. First, using changes in QOL provided a uniform method of measuring patient outcomes between multiple independent trials treating two different diseases. This enabled us to both conduct meta-analyses between disease areas and increase our statistical confidence in the conclusions. Secondly, the placebo response is more pronounced in subjective symptom measurements, such as those recorded in QOL questionnaires than in objective measurements [2, 22], so we expected to see greater differentiation between placebo responders and non-responders in these measurements. However, we also conducted secondary analyses using physiological clinical endpoints to validate the applicability of the findings to the trial design. For RA trials, the physiological clinical endpoint was an improvement of at least 20% in the American College of Rheumatology score (ACR20) and for Asthma trials, an above median change in the Forced Expiratory Volume (FEV_1_), a measure of lung volume frequently recorded in Asthma trials.

## Results and discussion

Our primary aim was to investigate the effect of previously reported placebo-associated genetic variants on patient outcomes in the placebo arms of Phase III trials for Asthma and RA. To address this question, we analyzed 784 patients from nine Phase III trials. Of those, 364 had been diagnosed with active RA and participated in one of five Phase III trials investigating Tocilizumab, a humanized monoclonal antibody targeting the Interleukin 6 receptor (IL6R). The other 420 patients were diagnosed with moderate to severe Asthma and participated in one of four trials investigating Lebrikizumab, a humanized monoclonal antibody targeting Interleukin 13 (IL13) (Table 1). Because it has been demonstrated that the placebo response is most evident in patient-reported assessments of their symptoms (e.g., QOL questionnaires) rather than in clinician-measured outcomes such as ACR20 or FEV_1_ [2, 22], we defined placebo response as a patient-reported improvement of symptoms in disease-specific QOL questionnaires. Furthermore, using the QOL questionnaires for phenotype definitions enabled direct comparisons between trials and between disease areas.

**Table 1:** Patient overview

	Study Name	# Placebo patients	Age distribution	Males:Females	End Trial R:NR	Early Trial R:NR	Reference
Rheumatoid arthritis	Option	63	*x̅* = 52.9, *σ* = 11.2, min = 24, max = 80	16:47	42:41	25:38	Smolen et al. [15]
	Lithe	111	*x̅* = 54.3, *σ* = 11.5, min = 20, max = 78	23:88	53:58	35:76	Kremer et al. [16]
	Ambition	27	*x̅* = 51.4, *σ* = 13.6, min = 26, max = 82	10:17	18:9	9:18	Jones et al. [17]
	Radiate	53	*x̅* = 51.0, *σ* = 13.1, min = 20, max = 74	12:41	21:32	20:33	Emery et al. [18]
	Toward	110	*x̅* = 57.0, *σ* = 11.0, min = 26, max = 80	34:86	60:50	43:67	Genovese et al. [19]
	TOTAL	364	*x̅* = 54.2, *σ* = 11.9, min = 20, max = 82	95:269	194:170	132:232	
Asthma	Lute	31	*x̅* = 52.8, *σ* = 15.5, min = 22, max = 75	11:20	7:24	8:23	Hanania et al. [20]
	Verse	19	*x̅* = 56.8, *σ* = 9.3, min = 39, max = 74	10:9	6:13	5:14	Hanania et al. [20]
	Lavolta 1	187	*x̅* = 51.6, *σ* = 11.8, min = 18, max = 75	70:117	38:149	56:131	Hanania et al. [21]
	Lavolta 2	183	*x̅* = 49.5, *σ* = 12.9, min = 18, max = 74	71:112	42:141	44:139	Hanania et al. [21]
	TOTAL	420	*x̅* = 51.0, *σ* = 12.6, min = 18, max = 75	162:258	93:327	113:307	
TOTAL		784	*x̅* = 52.5, *σ* = 12.3, min = 18, max = 82	257:527	287:497	245:539	

Demographic information for the 784 patients included in the study. Each patient was enrolled in one of nine clinical trials for the treatment of uncontrolled Asthma or Rheumatoid Arthritis. “End trial” refers to phenotype assignment of response (R) or non-response (NR) at the clinically defined conclusion of the trial. “Early trial” refers to the phenotype being defined as R/NR in the first month of treatment. References are for the original placebo-controlled study publication

We initially focused on the changes in symptoms relative to the baseline at the conclusion of each trial to maximize the relevancy of our findings to trial design. Using a logistic regression approach for identifying the genetic associations while accounting for potentially confounding variables, followed by random-effect model meta-analysis to combine our results from the two disease areas, we tested each previously reported autosomal placebo-associated variant for significant association with patients who reported an improvement of symptoms versus no change or worsening of symptoms (see “Patients and methods” section for discussion of phenotype assignment). We began our analyses by testing the 10 previously reported variants in autosomal genes involved in the four hypothesized placebo-implicated pathways (Table 2a). We were well powered to detect the associations in these variants with strong effects (Power = 0.86, assuming an effect size of 1.7, a minor allele frequency of c. 45%, which is comparable to rs4680, and the commonly accepted placebo response rate of 35% [23–25]). After Bonferroni correction for multiple hypothesis testing, none of the previously reported placebo-associated variants, including the well-characterized SNP rs4680, were significantly associated with patient-reported improvements at the conclusion of each trial in RA, Asthma, or a meta-analysis. In fact, most variants were well balanced between responders/non-responders (Supplemental Table 1). In the five RA trials, all variants shared similar allelic frequencies between the case/control groups. In Asthma, only two variants, rs4570625 from THP2 (*P* = 0.009, OR = 1.761) and rs6280 from DRD3 (*P* = 0.015, OR = 0.5933), showed a moderate, yet still statistically insignificant, difference. Notably, consistent with previous findings, Asthma patients homozygous for T/T (Ser/Ser) at rs6280 were enriched in the placebo responders group (59% vs. 47%), although this was not observed in RA patients [26]. On the other hand, Asthma patients homozygous for G/G at rs4570625 were more common in the non-responders group, an effect direction that is opposite to the previous findings [27]. Combined with the fact that neither of these two variants showed even moderate significance under a random-effect meta-analysis model or under association testing for physiological clinical endpoints (Supplemental Table 2), we conclude that these variants do not significantly impact the placebo response in these trials.

**Table 2:** Effect of previously reported variants on placebo-arm outcome

A. Response at the conclusion of the trial								
Pathway	Gene	SNP	Rheumatoid arthritis		Asthma		Meta-analysis	
			*P*	OR	*P*	OR	*P*(*R*)	OR(*R*)
Dopamine	COMT	rs4680	0.4904	1.118	0.9495	0.989	0.6390	1.058
	DBH	rs2873804	0.6423	0.929	0.7345	1.059	0.9161	0.988
	DRD3	rs6280	0.4189	0.872	0.0148	0.593	0.1075	0.735
	BDNF	rs6265	0.4131	1.184	0.3074	1.248	0.1950	1.214
Serotonin	TPH2	rs4570625	0.9836	1.004	0.0089	1.761	0.3299	1.315
	SLC6A4	rs4251417	0.4387	0.819	0.9683	0.990	0.5643	0.900
	HTR2A	rs2296972	0.1972	1.264	0.8436	1.041	0.2747	1.160
		rs622337	0.1678	1.282	0.9128	1.023	0.2685	1.162
Opioid	OPRM1	rs510769	0.9599	0.991	0.6785	1.085	0.8145	1.031
Endocannabinoid	FAAH	rs324420	0.9631	1.008	0.9102	0.976	0.9694	0.995

B. Placebo response in the first month of the trial								
Pathway	Gene	SNP	Rheumatoid arthritis		Asthma		Meta-analysis	
			*P*	OR	*P*	OR	*P*(*R*)	OR(*R*)
Dopamine	COMT	rs4680	0.9951	0.999	0.9311	1.015	0.9537	1.0068
	DBH	rs2873804	0.4087	1.144	0.4585	0.8897	0.9638	1.0057
	DRD3	rs6280	0.5616	1.105	0.0037	0.5571	0.4935	0.791
	BDNF	rs6265	0.8789	0.9686	0.9986	0.9996	0.9134	0.9841
Serotonin	TPH2	rs4570625	0.2054	1.267	0.2058	1.298	0.0740	1.2809
	SLC6A4	rs4251417	0.2478	1.348	0.6478	0.894	0.6773	1.0891
	HTR2A	rs2296972	0.255	1.228	0.4415	0.8604	0.8419	1.0361
		rs622337	0.242	1.232	0.4174	0.8524	0.8542	1.0344
Opioid	OPRM1	rs510769	0.0883	0.7355	0.9285	0.9833	0.2508	0.8465
Endocannabinoid	FAAH	rs324420	0.1314	1.316	0.2669	1.248	0.0626	1.2847

*P*-values and odds ratios for each variant’s logistic association with placebo response for each disease and a random-effect meta-analysis. (A) Placebo response observed at the conclusion of each trial, which varied by trial but ranged 24–52 weeks. (B) Placebo response recorded in the first month of treatment. A Bonferroni-corrected *P*-value threshold of 0.0025 was used to determine the significance. After correction, none of the previously reported variants showed significance in either disease or in the meta-analysis

Since we were unable to validate the previously reported variants, we hypothesized that the placebo effect might be a short-lived response and symptoms could return to normal or worsen over the long timeframe of Phase III trials (44–52 weeks). To test this hypothesis, we re-analyzed each previously reported variant, but redefined “placebo response” as a patient-reported improvement in symptoms during the first 4 weeks of the trial (percent change from baseline). Again, none of the previously reported variants showed a significant association with symptom improvement in the placebo arm (Table 2b, Supplemental Table 2). In Asthma, the two variants showing the lowest *P*-values in the later placebo response again showed the lowest *P*-values and same effect direction, although they still remained statistically insignificant. Interestingly, in RA, the *OPRM1* variant rs510769 showed a sharp decrease in *P*-value relative to the later time point, from 0.96 to 0.08, while maintaining an effect in the same direction. Taken together, these results suggest that the previously reported placebo-response-associated variants may not have a strong effect on patient outcomes in the placebo arms of immune-related disease trials.

Because the placebo-associated variants identified in studies investigating placebo treatments in pain and psychiatric disorders did not appear significantly associated with placebo-arm outcomes in immune-mediated disease trials, we sought to identify any novel variants that might have a strong effect under these disease conditions. We limited this analysis to patient response at the conclusion of each trial, as this timepoint is the most relevant to improving trial outcomes. We limited our analysis to common autosomal SNPs (MAF > 5%), and after QC and filtering, we tested 5,630,720 SNPs for RA, and 4,918,428 for Asthma. For each disease, we tested these SNPs for association with a placebo response while controlling for the potentially confounding effects of age, sex, population structure, and baseline disease severity. We observed no evidence of genomic inflation of the test statistic (inflation factors 1.047 and 1.024 for RA and Asthma, respectively) suggesting that the likely confounding factors were well accounted for. We also performed a random-effect meta-analysis of these results, where we analyzed the 4,697,728 shared SNPs. Although the study was well powered to detect common variants with strong effects (Power = 0.86, assuming an effect size of 1.7, a minor allele frequency of c. 45%, which is comparable to rs4680, and the commonly accepted placebo response rate of 35% [23–25]), we were unable to detect any genome-wide significant hits (Supplemental Fig. 1). In RA, the most significant hit was a haplotype block tagged by rs2613713 (*P* = 1.426e−06, OR = 2.363), which lies in an intergenic region on chromosome 8 (p21.3). However, this block became convincingly insignificant under the meta-analysis (*P*_R_ = 0.15, OR_R_ = 1.656). In Asthma patients, the most significant hit was rs6694886, an intronic G>T variant in *CAMTA1* (calmodulin binding transcription activator 1) on chromosome 1 (*P* = 2.529e−06, OR = 2.448). However, like with the most significant hit in RA, evidence vanished under the meta-analysis, dropping to *P*_R_ = 0.5948 with OR_R_ = 1.362. Because none of the limited “suggestive” variants were genome-wide significant (*P* < 10e−8), and because they lost statistical evidence under a random-effect meta-analysis, we are not confident that these are true placebo-associated variants. Rather, our results suggest that individual gene variants do not strongly affect the placebo response. However, it is important to note that due to the small sample size in this study, we are unable to rule out minor effects of single variants, or any effect that might arise from pathway-wide burdens.

In summary, our findings suggest that the biological origin of the placebo response is complex and likely variable between disease areas, and care must be taken when generalizing findings regarding the genetic causes of the placebo effect. Although previous studies have identified variants that influence the likelihood of response to placebo treatment for pain-related and psychiatric conditions, these variants appear to have very little, if any, effect on the experiences of placebo-arm patients in Phase III trials for immune-mediated diseases. Furthermore, although our study was moderately well powered, we were unable to detect any genome-wide significant variations between placebo responders and non-responders, supporting the conclusion that although genetics may play a role in the placebo effect, the role of genomic variation is small and likely overpowered by other, possibly non-genetic factors. Similar to previous studies, we are limited in drawing full conclusions by the lack of a no-treatment control. Unfortunately, however, we see little option for addressing this shortfall, as such a control group is not possible for diseases with existing acceptable treatment options.

Our study highlights the need for further research into the genetic basis of the placebo effect. It is likely that, like other complex traits, the placebo response is modulated by multiple variants with a relatively small effect each. In order to identify those variants, a larger sample size is likely to be required. We believe costs associated with obtaining the sequencing data would be justified, because a solid understanding of the variation affecting response under placebo treatment would allow its confounding influence could be accounted for in trial design, thus potentially leading to more efficient, less costly trials with higher success rates.

## Patients and methods

This analysis was performed on 784 non-related European patients from the placebo arms of nine clinical trials for RA and Asthma. Patients were 18–82 years old and the male:female ratio was 257:527 (Table 1). Genomic DNA was extracted from patients’ blood samples using the DNA Blood400 kit (Chemagic) and eluted in 50 µL Elution Buffer (EB, Qiagen). It was then sheared (Covaris LE220) and sequencing libraries were prepared using the TruSeq Nano DNA HT kit (Illumina Inc.). The whole-genome sequencing target coverage was 30×. Libraries were sequenced as 150-bp paired-end reads on the Illumina HiSeqX sequencer. Reads were mapped to hg38 using BWA-MEM [28] and variant calling was performed using GATK best practices [29]. Variant calling was performed using GATK HaplotypeCaller to generate sample level variant calls. The samples were then jointly genotyped separately for Asthma and RA samples, resulting in one VCF file for each disease area. To assure that only the most high-confidence variants are included in the association analysis, we set genotypes whose genotype quality was less than 20 to missing. We also removed variants with a missingness of more than 1%. Because our focus was on previously reported common variants and because of the limited size of our sample set, we chose to not include variants with an allele frequency of less than 5% in subsequent analyses.

The population structure was determined for patient cohorts from the joint genotyping SNPs from each disease (analyzed separately) using the GENESIS R package (version 2.6.1) [30]. GENESIS uses the PC-AiR method to determine population structure, a Principal-Components-based analysis that assesses cryptic relatedness from genome-wide sets of SNPs while accounting for potentially confounding relatedness [30]. GENESIS was also used to verify previous relatedness estimations, ensuring that all individuals included in the analyses were unrelated.

Each individual was determined to be a responder or a non-responder at two timepoints. We defined responders as those patients who reported improvement in disease severity, as assessed by the percent change from baseline in the appropriate QOL questionnaire, whereas non-responders were those patients who reported no change or a worsening of symptoms. For consistent comparison between disease areas, no minimum improvement cutoff was enforced. In other words, responders may display any amount of positive change in symptoms, even if minor. For RA, this response was recorded in the Health Assessment Questionnaire (HAQ), and for Asthma, the Asthma Quality of Life Questionnaire (AQLQ). A placebo response greater than zero was observed in all trials, and in most cases the placebo response exceeded the minimal clinically important difference (MCID). For example, the AQLQ changes for the placebo arms of LAVOLTA 1 and 2 were 0.78 and 0.80, respectively, which exceed the MCID of 0.5^18^.

Using QOL questionnaires provided two benefits over other recorded values: first, placebo responses are most evident in patient-reported symptom assessments [2, 22], and second it represents the most consistent measurement of outcome between different trials and disease areas. In the cases where a value was absent, we used a last observation carried forward (LOCF) approach to fill in missing values. This approach was most notably applied in the Lute and Verse trials, as the early discontinuation of these trials resulted in those patients enrolled later missing data at the specified endpoint. Each individual was assigned two phenotypes based on timeframe of comparison to baseline, where an early response refers to a reported improvement in symptoms in the first month of the trial (assessed at week 4), and late responders were those who reported an improvement at the conclusion of the trial (week 24 for RA trials and week 52 for Asthma trials).

To identify the variants associated with placebo response, we used PLINK (1.90b4.4) to perform logistic regression genetic association analysis contrasting placebo responders and non-responders [31–33]. Age, sex, baseline disease severity (baseline clinical measure of interest, e.g., AQLQ/HAQ), population structure (first five principal components from GENESIS), and trial (as dummy variable) were included as covariates to account for confounding influences such as imbalance and regression to the mean. Our approach assumed additive genetic effects, i.e., one alternate allele has a bigger effect than none, and that two have a bigger effect than one. In total, three rounds of association studies were performed; first, only the previously reported placebo-associated autosomal SNPs were tested at two time-points (conclusion of the trial and first month of treatment), and later a genome-wide set of common (MAF >5%) autosomal SNPs were investigated (only at the conclusion of the trial). Association for each disease was performed separately. Following disease-specific association analysis, a meta-analysis (--meta flag in PLINK) combining the two diseases areas was performed using the same version of PLINK to determine those variants with generalized placebo-response-associated phenotypes [34]. Because placebo response rates varied between diseases, only the more conservative random-effect model was interpreted.

## Electronic supplementary material

Supplemental Figure 1(DOCX 348 kb)

Supplemental Tables 1 and 2(DOCX 94 kb)

## References

[1] Miller FG, Kaptchuk TJ (2008). The power of context: reconceptualizing the placebo effect. J R Soc Med.

[2] Finniss DG, Kaptchuk TJ, Miller F, Benedetti F (2010). Biological, clinical, and ethical advances of placebo effects. Lancet.

[3] Colagiuri B, Schenk LA, Kessler MD, Dorsey SG, Colloca L (2015). The placebo effect: from concepts to genes. Neuroscience.

[4] Grignolo A, Pretorius S. Phase III trial failures: costly, but preventable. Appl Clin Trials. 2016;25. http://www.appliedclinicaltrialsonline.com/phase-iii-trial-failures-costly-preventable.

[5] Scannell JW, Blanckley A, Boldon H, Warrington B (2012). Diagnosing the decline in pharmaceutical R&D efficiency. Nat Rev Drug Discov.

[6] Mullin R. Tufts study finds big rise in cost of drug development | chemical & engineering news. Chem Eng News. 2014. http://cen.acs.org/articles/92/web/2014/11/Tufts-Study-Finds-Big-Rise.html. Accessed 7 Aug 2017.

[7] Hall KT, Loscalzo J, Kaptchuk TJ (2015). Genetics and the placebo effect: the placebome. Trends Mol Med.

[8] Kaptchuk TJ, Miller FG (2015). Placebo effects in medicine. N Engl J Med.

[9] Holmes RD, Tiwari AK, Kennedy JL (2016). Mechanisms of the placebo effect in pain and psychiatric disorders. Pharmacogenomics J.

[10] Hall KT, Lembo AJ, Kirsch I, Ziogas DC, Douaiher J, Jensen KB (2012). Catechol-O-methyltransferase val158met polymorphism predicts placebo effect in irritable bowel syndrome. PLoS ONE.

[11] Servick K (2014). Outsmarting the placebo effect. Science.

[12] Butler C, Steptoe A (1986). Placebo responses: an experimental study of psychophysiological processes in asthmatic volunteers. Br J Clin Psychol.

[13] Kemeny ME, Rosenwasser LJ, Panettieri RA, Rose RM, Berg-Smith SM, Kline JN (2007). Placebo response in asthma: a robust and objective phenomenon. J Allergy Clin Immunol.

[14] Abdullah N (2015). Placebo effect in the treatment of rheumatoid arthritis: a systematic review and meta-analysis of randomized controlled trials. Ann Rheum Dis.

[15] Smolen JS, Beaulieu A, Rubbert-Roth A, Ramos-Remus C, Rovensky J, Alecock E (2008). Effect of interleukin-6 receptor inhibition with tocilizumab in patients with rheumatoid arthritis (OPTION study): a double-blind, placebo-controlled, randomised trial. Lancet.

[16] Kremer JM, Blanco R, Brzosko M, Burgos-Vargas R, Halland AM, Vernon E (2011). Tocilizumab inhibits structural joint damage in rheumatoid arthritis patients with inadequate responses to methotrexate: results from the double-blind treatment phase of a randomized placebo-controlled trial of tocilizumab safety and prevention of structural joint damage at one year. Arthritis Rheum.

[17] Jones G, Sebba A, Gu J, Lowenstein MB, Calvo A, Gomez-Reino JJ (2010). Comparison of tocilizumab monotherapy versus methotrexate monotherapy in patients with moderate to severe rheumatoid arthritis: the AMBITION study. Ann Rheum Dis.

[18] Emery P, Keystone E, Tony HP, Cantagrel A, van Vollenhoven R, Sanchez A (2008). IL-6 receptor inhibition with tocilizumab improves treatment outcomes in patients with rheumatoid arthritis refractory to anti-tumour necrosis factor biologicals: results from a 24-week multicentre randomised placebo-controlled trial. Ann Rheum Dis.

[19] Genovese MC, McKay JD, Nasonov EL, Mysler EF, da Silva NA, Alecock E (2008). Interleukin-6 receptor inhibition with tocilizumab reduces disease activity in rheumatoid arthritis with inadequate response to disease-modifying antirheumatic drugs: the tocilizumab in combination with traditional disease-modifying antirheumatic drug therapy study. Arthritis Rheum.

[20] Hanania NA, Noonan M, Corren J, Korenblat P, Zheng Y, Fischer SK (2015). Lebrikizumab in moderate-to-severe asthma: pooled data from two randomised placebo-controlled studies. Thorax.

[21] Hanania NA, Korenblat P, Chapman KR, Bateman ED, Kopecky P, Paggiaro P (2016). Efficacy and safety of lebrikizumab in patients with uncontrolled asthma (LAVOLTA I and LAVOLTA II): replicate, phase 3, randomised, double-blind, placebo-controlled trials. Lancet Respir Med.

[22] Wechsler ME, Kelley JM, Boyd IOE, Dutile S, Marigowda G, Kirsch I (2011). Active albuterol or placebo, sham acupuncture, or no intervention in asthma. N Engl J Med.

[23] Beecher HK (1955). The powerful placebo. J Am Med Assoc.

[24] Johnson JL. GAS Power Calculator. 2017. http://csg.sph.umich.edu/abecasis/cats/gas_power_calculator/reference.html. Accessed 9 Aug 2017.

[25] Skol AD, Scott LJ, Abecasis GR, Boehnke M (2006). Joint analysis is more efficient than replication-based analysis for two-stage genome-wide association studies. Nat Genet.

[26] Bhathena A, Wang Y, Kraft JB, Idler KB, Abel SJ, Holley-Shanks RR (2013). Association of dopamine-related genetic loci to dopamine D3 receptor antagonist ABT-925 clinical response. Transl Psychiatry.

[27] Furmark T, Appel L, Henningsson S, Ahs F, Faria V, Linnman C (2008). A link between serotonin-related gene polymorphisms, amygdala activity, and placebo-induced relief from social anxiety. J Neurosci.

[28] Li H. Aligning sequence reads, clone sequences and assembly contigs with BWA-MEM. ArXiv13033997 Q-Bio. 2013. http://arxiv.org/abs/1303.3997.

[29] GATK | Best Practices. https://software.broadinstitute.org/gatk/best-practices/. Accessed 6 Sep 2017.

[30] Conomos MP, Thornton T, Gogarten SM. GENetic EStimation and Inference in Structured samples (GENESIS): statistical methods for analyzing genetic data from samples with population structure and/or relatedness. 2017. http://bioconductor.org/packages/GENESIS/.

[31] Purcell S, Chang C. PLINK v.1.90b4.4. www.cog-genomics.org/plink/1.9/.

[32] Chang CC, Chow CC, Tellier LC, Vattikuti S, Purcell SM, Lee JJ (2015). Second-generation PLINK: rising to the challenge of larger and richer datasets. Gigascience.

[33] Hill A, Loh PR, Bharadwaj RB, Pons P, Shang J, Guinan E (2017). Stepwise distributed open innovation contests for software development: acceleration of genome-wide association analysis. Gigascience.

[34] Willer CJ, Li Y, Abecasis GR (2010). METAL: fast and efficient meta-analysis of genomewide association scans. Bioinformatics.

