# Supplementary material for: Previously reported placebo-response-associated variants do not predict patient outcomes in inflammatory disease Phase III trial placebo arms
Source: Genes Immun. 2018 Mar 18;20(2):172–9. doi: 10.1038/s41435-018-0018-z (PMC6515143; doi:10.1038/s41435-018-0018-z)
Supplement: Supplementary file 1 — Supplemental Figure 1(DOCX 348 kb) [file 41435_2018_18_MOESM1_ESM.docx]

**
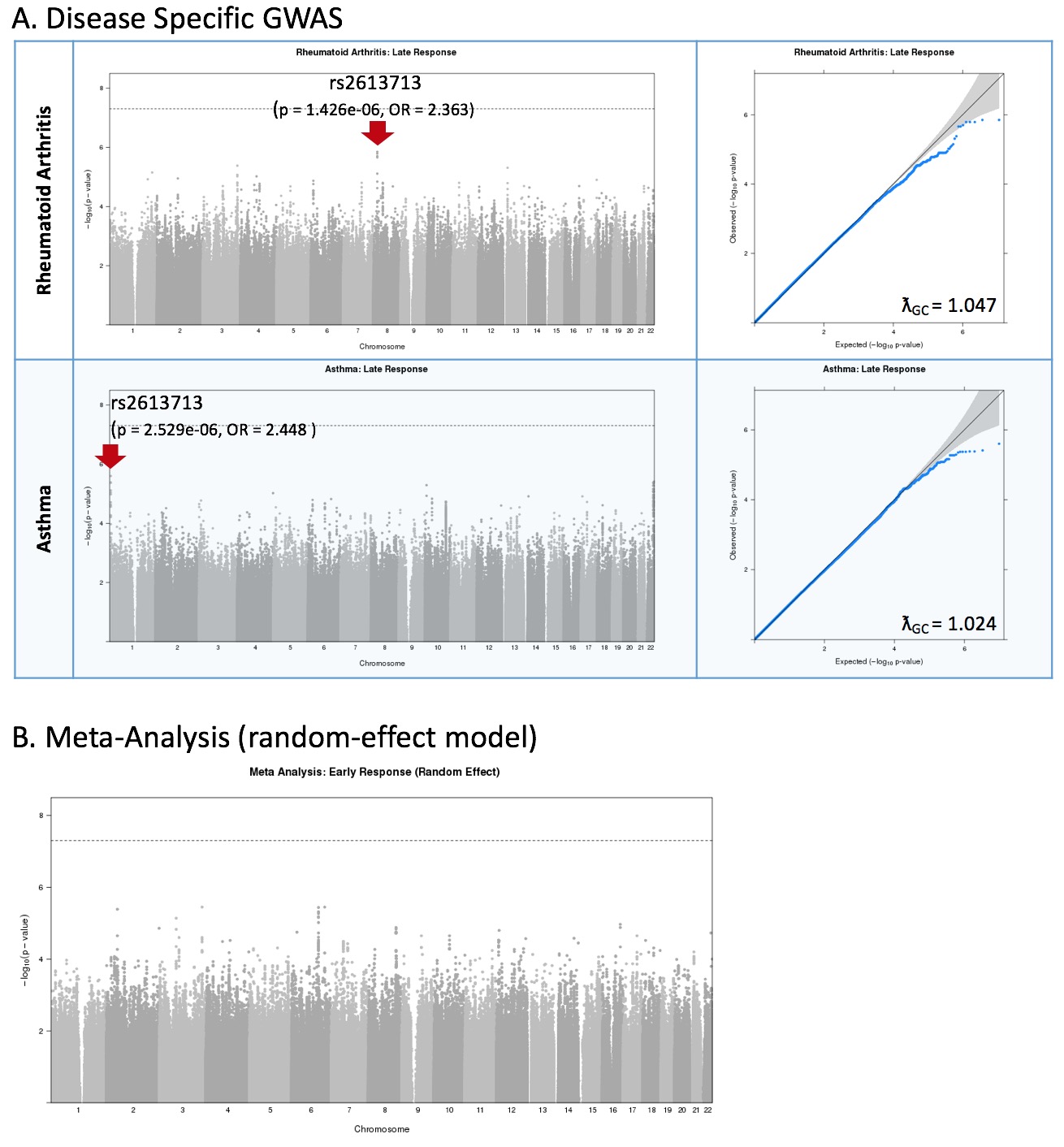
Supplemental Figure 1: *GWAS results for placebo-arm outcome.***

Manhattan plot representations of GWAS analysis results for SNP associations with placebo response. Results from the clinical endpoint are shown as they are the most relevant to improving trial design. Logistic associations were performed and covariate controlled for age, sex, population structure, and baseline disease severity. (A) GWAS results, presented as a Manhattan plot and QQ plot (for QA), for RA and Asthma analyses. For each disease, the “most significant” hit is indicated by a red arrow. QQ plots and genomic inflation factors show that covariates were well controlled. (B) Manhattan plot of random-effect meta-analysis. No hits passed a genome-wide significance threshold of 5×10^-8^.
