# Supplementary material for: Previously reported placebo-response-associated variants do not predict patient outcomes in inflammatory disease Phase III trial placebo arms
Source: Genes Immun. 2018 Mar 18;20(2):172–9. doi: 10.1038/s41435-018-0018-z (PMC6515143; doi:10.1038/s41435-018-0018-z)
Supplement: Supplementary file 2 — Supplemental Tables 1 and 2(DOCX 94 kb) [file 41435_2018_18_MOESM2_ESM.docx]

**Supplemental Table 1: *Variant overview.***

Overview of previously reported variants. All previously-reported variants were tested for both conditions. (A) SNP alleles for each previously reported placebo-associated variant. Original indication refers to the condition in which the original placebo association was identified. (B) Allelic frequencies of each SNP in clinical endpoint responder and non-responder groups. (C) Allelic frequencies of each SNP in first month responder/non-responder groups.

| **A. SNP Information** | | | | | |
| --- | --- | --- | --- | --- | --- |
| **Pathway** | **Gene** | **SNP** | **Original Indication** | **Allele B** | **Allele B** |
| Dopamine | COMT | rs4680 | IBS, healthy volunteers | A | G |
|  | DBH | rs2873804 | depression | C | T |
|  | DRD3 | rs6280 | schizophrenia | C | T |
|  | BDNF | rs6265 | healthy volunteers | T | C |
| Serotonin | TPH2 | rs4570625 | social anxiety disorder | T | G |
|  | SLC6A4 | rs4251417 | depression | T | C |
|  | HTR2A | rs2296972 | depression | A | C |
|  |  | rs622337 | depression | G | A |
| Opioid | OPRM1 | rs510769 | healthy volunteers | T | C |
| Endocannabanoid | FAAH | rs324420 | healthy volunteers | A | C |

| **B. Allelic Frequencies, Placebo Response at Primary Clinical Endpoint** | | | |  |  |  |  |  |  |
| --- | --- | --- | --- | --- | --- | --- | --- | --- | --- |
| **Pathway** | **Gene** | **SNP** |  | **Rheumatoid Arthritis** | | | **Asthma** | | |
|  |  |  | **Group** | **AA** | **AB** | **BB** | **AA** | **AB** | **BB** |
| Dopamine | COMT | rs4680 | Responder | 0.200 | 0.569 | 0.231 | 0.204 | 0.548 | 0.247 |
|  |  |  | Non-Responder | 0.206 | 0.506 | 0.288 | 0.254 | 0.483 | 0.263 |
|  | DBH | rs2873804 | Responder | 0.185 | 0.554 | 0.262 | 0.247 | 0.430 | 0.323 |
|  |  |  | Non-Responder | 0.241 | 0.447 | 0.312 | 0.248 | 0.422 | 0.330 |
|  | DRD3 | rs6280 | Responder | 0.082 | 0.426 | 0.492 | 0.032 | 0.376 | 0.591 |
|  |  |  | Non-Responder | 0.112 | 0.429 | 0.459 | 0.076 | 0.450 | 0.474 |
|  | BDNF | rs6265 | Responder | 0.026 | 0.349 | 0.626 | 0.032 | 0.355 | 0.613 |
|  |  |  | Non-Responder | 0.029 | 0.312 | 0.659 | 0.046 | 0.315 | 0.639 |
| Serotonin | TPH2 | rs4570625 | Responder | 0.051 | 0.333 | 0.615 | 0.065 | 0.441 | 0.495 |
|  |  |  | Non-Responder | 0.071 | 0.335 | 0.594 | 0.021 | 0.358 | 0.621 |
|  | SLC6A4 | rs4251417 | Responder | 0.010 | 0.133 | 0.856 | 0.011 | 0.269 | 0.720 |
|  |  |  | Non-Responder | 0.029 | 0.147 | 0.824 | 0.015 | 0.229 | 0.755 |
|  | HTR2A | rs2296972 | Responder | 0.097 | 0.395 | 0.508 | 0.054 | 0.441 | 0.505 |
|  |  |  | Non-Responder | 0.041 | 0.396 | 0.562 | 0.067 | 0.394 | 0.538 |
|  |  | rs622337 | Responder | 0.097 | 0.395 | 0.508 | 0.054 | 0.430 | 0.516 |
|  |  |  | Non-Responder | 0.047 | 0.382 | 0.571 | 0.064 | 0.391 | 0.544 |
| Opioid | OPRM1 | rs510769 | Responder | 0.067 | 0.397 | 0.536 | 0.075 | 0.398 | 0.527 |
|  |  |  | Non-Responder | 0.101 | 0.369 | 0.530 | 0.064 | 0.382 | 0.554 |
| Endo-cannabanoid | FAAH | rs324420 | Responder | 0.072 | 0.323 | 0.605 | 0.043 | 0.344 | 0.613 |
|  |  |  | Non-Responder | 0.071 | 0.329 | 0.600 | 0.040 | 0.339 | 0.621 |

| **C. Allelic Frequencies, Placebo Response in First Month of Trial** | | | |  |  |  |  |  |  |
| --- | --- | --- | --- | --- | --- | --- | --- | --- | --- |
| **Pathway** | **Gene** | **SNP** |  | **Rheumatoid Arthritis** | | | **Asthma** | | |
|  |  |  | **Group** | **AA** | **AB** | **BB** | **AA** | **AB** | **BB** |
| Dopamine | COMT | rs4680 | Responder | 0.203 | 0.541 | 0.256 | 0.230 | 0.531 | 0.239 |
|  |  |  | Non-Responder | 0.203 | 0.539 | 0.259 | 0.257 | 0.485 | 0.257 |
|  | DBH | rs2873804 | Responder | 0.218 | 0.534 | 0.248 | 0.195 | 0.487 | 0.319 |
|  |  |  | Non-Responder | 0.207 | 0.487 | 0.306 | 0.267 | 0.401 | 0.332 |
|  | DRD3 | rs6280 | Responder | 0.105 | 0.429 | 0.466 | 0.053 | 0.336 | 0.611 |
|  |  |  | Non-Responder | 0.091 | 0.427 | 0.483 | 0.072 | 0.469 | 0.459 |
|  | BDNF | rs6265 | Responder | 0.023 | 0.331 | 0.647 | 0.027 | 0.327 | 0.646 |
|  |  |  | Non-Responder | 0.030 | 0.332 | 0.638 | 0.049 | 0.322 | 0.629 |
| Serotonin | TPH2 | rs4570625 | Responder | 0.068 | 0.353 | 0.579 | 0.018 | 0.451 | 0.531 |
|  |  |  | Non-Responder | 0.056 | 0.323 | 0.621 | 0.036 | 0.349 | 0.616 |
|  | SLC6A4 | rs4251417 | Responder | 0.015 | 0.180 | 0.805 | 0.027 | 0.221 | 0.752 |
|  |  |  | Non-Responder | 0.022 | 0.116 | 0.862 | 0.010 | 0.244 | 0.746 |
|  | HTR2A | rs2296972 | Responder | 0.113 | 0.368 | 0.519 | 0.044 | 0.407 | 0.549 |
|  |  |  | Non-Responder | 0.048 | 0.411 | 0.541 | 0.072 | 0.404 | 0.524 |
|  |  | rs622337 | Responder | 0.113 | 0.368 | 0.519 | 0.044 | 0.398 | 0.558 |
|  |  |  | Non-Responder | 0.052 | 0.401 | 0.547 | 0.068 | 0.401 | 0.531 |
| Opioid | OPRM1 | rs510769 | Responder | 0.068 | 0.331 | 0.602 | 0.071 | 0.381 | 0.549 |
|  |  |  | Non-Responder | 0.092 | 0.415 | 0.493 | 0.065 | 0.388 | 0.547 |
| Endo-cannabanoid | FAAH | rs324420 | Responder | 0.075 | 0.361 | 0.564 | 0.044 | 0.372 | 0.584 |
|  |  |  | Non-Responder | 0.069 | 0.306 | 0.625 | 0.039 | 0.329 | 0.632 |

**Supplemental Table 2: *Association with physiological clinical endpoints.***

P-values and odds ratios for each variant’s logistic association with placebo response for each disease. Placebo response in this table was defined as ACR20 for RA trials and as changes in FEV_1_ for Asthma trials. Refer to main article for details.

| **Pathway** | **Gene** | **SNP** | **Rheumatoid Arthritis** | | **Asthma** | |
| --- | --- | --- | --- | --- | --- | --- |
|  |  |  | **P** | **OR** | **P** | **OR** |
| Dopamine | COMT | rs4680 | 0.5160 | 1.129 | 0.5240 | 1.008 |
|  | DBH | rs2873804 | 0.6090 | 1.099 | 0.0764 | 1.253 |
|  | DRD3 | rs6280 | 0.3538 | 0.832 | 0.1569 | 1.238 |
|  | BDNF | rs6265 | 0.2088 | 1.333 | 0.8851 | 0.986 |
| Serotonin | TPH2 | rs4570625 | 0.3898 | 0.827 | 0.5295 | 0.900 |
|  | SLC6A4 | rs4251417 | 0.6808 | 0.881 | 0.7889 | 1.054 |
|  | HTR2A | rs2296972 | 0.2218 | 1.278 | 0.4729 | 0.897 |
|  |  | rs622337 | 0.1315 | 1.350 | 0.4238 | 0.885 |
| Opioid | OPRM1 | rs510769 | 0.4414 | 1.162 | 0.2864 | 1.176 |
| Endocannabinoid | FAAH | rs324420 | 0.7261 | 0.930 | 0.0880 | 0.757 |
